# Bioengineered Skin Substitutes: The Role of Extracellular Matrix and Vascularization in the Healing of Deep Wounds

**DOI:** 10.3390/jcm8122083

**Published:** 2019-12-01

**Authors:** Francesco Urciuolo, Costantino Casale, Giorgia Imparato, Paolo A. Netti

**Affiliations:** 1 Department of Chemical, Materials and Industrial Production Engineering (DICMAPI) University of Naples Federico II, P.le Tecchio 80, 80125 Naples, Italy; costantino.casale@unina.it (C.C.); nettipa@unina.it (P.A.N.); 2 Interdisciplinary Research Centre on Biomaterials (CRIB), University of Naples Federico II P.le Tecchio 80, 80125 Naples, Italy; 3 Center for Advanced Biomaterials for HealthCare@CRIB, Istituto Italiano di Tecnologia, Largo Barsanti e Matteucci 53, 80125 Naples, Italy; giorgia.imparato@iit.it

**Keywords:** skin substitutes, tissue engineering, wound healing, extracellular matrix, bottom-up tissue engineering, vascularization, bioreactors, dermal substitutes, scar tissue

## Abstract

The formation of severe scars still represents the result of the closure process of extended and deep skin wounds. To address this issue, different bioengineered skin substitutes have been developed but a general consensus regarding their effectiveness has not been achieved yet. It will be shown that bioengineered skin substitutes, although representing a valid alternative to autografting, induce skin cells in repairing the wound rather than guiding a regeneration process. Repaired skin differs from regenerated skin, showing high contracture, loss of sensitivity, impaired pigmentation and absence of cutaneous adnexa (i.e., hair follicles and sweat glands). This leads to significant mobility and aesthetic concerns, making the development of more effective bioengineered skin models a current need. The objective of this review is to determine the limitations of either commercially available or investigational bioengineered skin substitutes and how advanced skin tissue engineering strategies can be improved in order to completely restore skin functions after severe wounds.

## 1. Introduction

**Figure 1:**
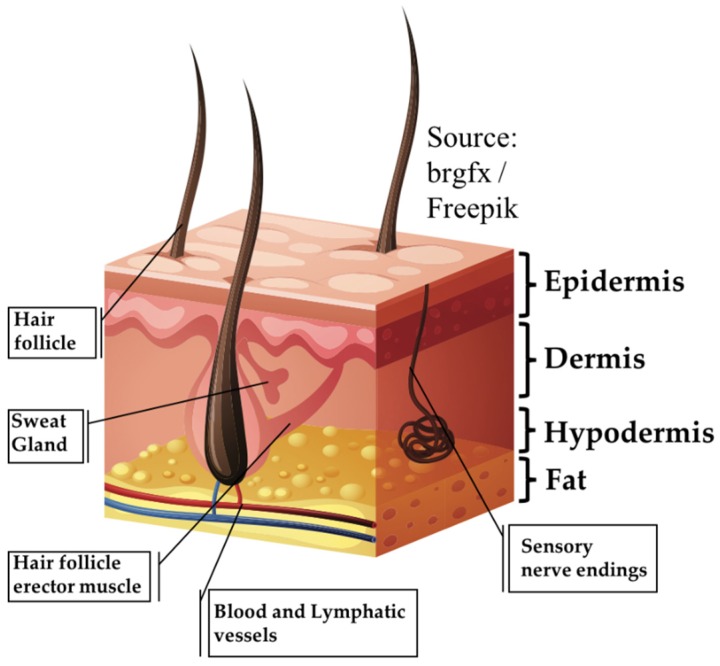
Main components of the human skin. (Image source: brgfx/Freepik).

**Figure 2:**
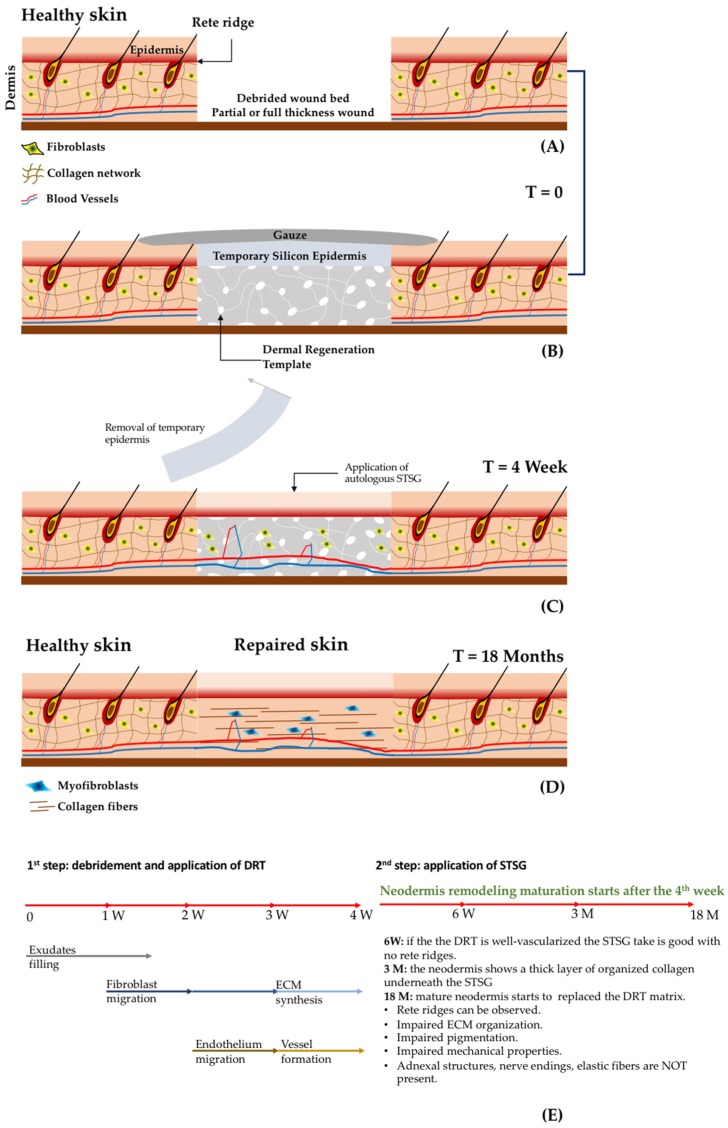
The main steps of a two-step procedure to treat deep and partial wounds with the application of a DRT. (**A**) Healthy skin and wound bed after debridement. (**B**) Application of a DRT possessing an artificial silicone epidermis and covered with gauze. (**C**) Removal of the silicone epidermis and application of the STSG. (**D**) Long-term appearance of the repaired dermis. (**E**) Cellular end extracellular dynamics occurring during the wound healing process after the application of a DRT. W = week; M = month. DRT, dermal regeneration templates; STSG, split thickness skin graft; ECM, extracellular matrix.

The skin is the largest organ of the body, accounting for about 15% of the total adult body weight. It is made up of three layers: the epidermis, dermis, and the hypodermis (Figure 1, [1]). Bioengineered skin substitutes, in the form of either cellularized engineered skin grafts or acellular dermal regeneration templates (DRT), have been developed to address two main issues still affecting the repair of extended deep wounds [2,3,4,5,6,7,8,9,10]: firstly, limiting the amount of healthy skin removed from the patient needed for closure; and secondly, acting as promoter for the restoration of the physiologic conditions of the skin avoiding the formation of severe scars. Skin acts not only as a barrier between the organism and the environment preventing invasion of pathogens and fending off chemical and physical assaults [11], it also plays a crucial role in the regulation of body temperature, moisture, and trafficking of water and solutes [12]. In addition, the sensory system of the skin allows the sensing of pain, temperature, light touch, discriminative touch, vibration and pressure. Finally, other important adnexal structures, such as sweat glands and hair follicles, contribute to the functionality of the healthy skin. In addition to the epidermis, severe damage due to burns, chronic ulcers and reconstructive surgeries, induce the destruction of the dermis. Unlike the epidermis, the dermis is characterized by an impaired healing process in which the final assembly of the extracellular matrix (ECM) is far from physiologic conditions. This mismatch compromises the reestablishment of the aforementioned regulatory functions of the whole organ [13,14,15]. The components of the ECM of the dermis (collagen elastin, hyaluronic acid, fibronectin, perlacan, water and other molecules), possess specific three-dimensional arrangements of sequences orchestrating the cross-talk among the different cell populations comprising the skin. Ultimately, such cross-talk affects the attachment, migration, differentiation and morphogenetic phenomena. For instance, the ECM promotes ‘appropriate’ communications between keratinocytes and the fibroblasts, and it is responsible for the formation and maintenance of the adnexal structures such as hair follicles, sweat glands and innervations [16,17,18]. Furthermore, when such adnexal structures become compromised, the self-regeneration of the epidermis cannot occur, and the wound becomes hard to heal. The repair of a deep wound can be divided into four subsequent phases [19,20]: (i) the coagulation and homeostasis phase (immediately after injury); (ii) the inflammatory phase (shortly after injury to tissue), during which swelling takes place; (iii) the proliferation period, where new tissues and blood vessels are formed; and (iv) the maturation phase, in which remodeling of new tissues takes place. How the maturation phase takes place determines the difference between repair and regeneration. The former is a “mere” closure process where fibroblasts bridge the wound gap by organizing their ECM differently from the healthy status. The latter restores the organization of the ECM that will appear indistinguishable from the healthy status [21]. The impaired ECM organization featuring the repair process depresses the regulatory and repository role of the extracellular space that ultimately forms an extended scar characterized by the loss of biological functionalities, inducing the insurgence of severe aesthetics and mobility-associated concerns. A classical approach to skin grafting and repairing is depicted in Figure 2. After debridement of the wound bed, a DRT is applied. Fibroblasts and endothelial cells from the recipient take at least one month to invade the DRT. After this time, it is possible to apply a split thickness skin graft (STSG): an epidermis with a layer of dermis removed from healthy sites of the patient. Both vascularization and fibroblast-secreted ECM molecules affect the take of the STSG that serves to trigger the regeneration of the epidermis due to the lack of adnexa and basal lamina [22,23,24,25,26]. As shown in Figure 2, with a period of two years, the remodeling of the neodermis occurs. To date, even though progress in biomaterials science and tissue engineering has led to the realization of different classes of skin substitutes (either cellularized or not), their healing potential is still limited in triggering a repair process instead of regeneration. In addition to economics, safety and regulatory (in case of allogenic or xenogeneic materials) concerns, in this work the currently available skin substitutes will be reviewed in the light of the composition of the dermis compartment and how this can affect the regeneration process. DRT can be fabricated starting from connective tissues of either allogenic or xenogeneic origin after removal of the cellular component [27,28]. The decellularization processes remove the associated risk of transmission of pathogens, preserving the composition of the ECM. On the other hand, the functionality of molecules of the ECM resulting from the decellularization processes compromise the correct signal presentation to the cells [16,17,18]. Pre-cellularization with endothelial cells, fibroblasts and keratinocytes seems to improve the biological performance of reconstructed three-dimensional matrices of both natural or synthetic origins [2,27,29,30,31], by speeding up the vascularization, the synthesis of neodermis, the take of the STSG and the closure of the wounds [32,33,34]. Nevertheless, the reconstructed three-dimensional matrices used to accommodate living cells prior to the implant are composed of exogenous biomatrices that possess composition, stiffness and three-dimensional arrangements that are quite different from the native dermis. For this reason, some doubts on their effectiveness in triggering a regeneration process have been raised [17]. Finally, a tissue engineering strategy that use patients’ own cells to build up in vitro a human-like vascularized ECM featured by the absence of any exogenous material, is presented as an alternative to guide the wound toward a physiological regeneration process [35,36,37]. 

## 2. Tissue Engineering Strategies for Skin Regeneration 

Tissue engineering aims at developing strategies to allow tissue and organ regeneration [32] by two approaches: (i) In in vitro tissue engineering the patient’s human skin is re-built in a laboratory using either endogenous or allogenic cell lines (keratinocytes and fibroblasts); after a period of cultivation in three-dimensional matrices [24] and bioreactors [10,38], the engineered skin is then implanted [32]; (ii) in in vivo tissue engineering a three-dimensional matrix is introduced in the wound bed; such matrices are bio-functionalized in order to attract both cells and growth factors supporting skin regeneration [38]. The use of de novo fabricated skin becomes necessary when skin self-regeneration is hindered by adverse conditions [10]; in particular, when severe burns (second-, third-, and fourth-degree burns), chronic ulcers, surgery or trauma lead to the destruction of the dermis and underlying tissues (fat, muscle or bone) are exposed [39,40]. Bioengineered skin substitutes can be classified according to the following categories.

- Cellularized epithelial tissues: used for superficial wounds, when the dermis is not (or is partially) damaged; autologous, allogenic or xenogeneic epithelial tissues are cultured in vitro and then implanted. In this case, the application of an STSG is not required. Engineered epithelial tissues are in general formed by cell sheets two or three cell layers thick [41,42].
- Cellularized dermis: when the dermis is damaged, autologous or allogenic fibroblasts are embedded in a three-dimensional matrix and then implanted in the wound bed. This procedure implies a second surgical step for the application of an epithelial layer using an STSG as shown in Figure 2 [41,42].
- Cellularized composite skin (or full thickness): engineered tissues containing both epithelial and dermal tissues. They are composed of epithelial tissue grown on a dermis surrogate composed of fibroblasts entrapped in a biomaterial [4,41,42,43]. Due to the presence of an epidermis layer, the application of an STSG can be avoided.
- Acellular dermal substitutes: or derma regeneration templates (DRT) that are porous 3D biomaterials (non-containing cells) applied in the missing dermis after wound debridement. This procedure implies a second surgical step for the application of an epithelial layer coming from an STSG, as shown in Figure 2 [4,41,42,43].

### 2.1. Dermal Regeneration Templates (DRT): Materials and Fabrication Techniques 

Porous and fibrous materials. Regardless of the tissue engineering strategy used (in vitro vs. in vivo approaches), a 3D scaffold supporting cell growth is required [42]. Scaffolds are biomaterials acting as temporary porous structures mimicking the 3D architecture of human tissue. In the case of skin tissue engineering, the tissue that one would like to mimic is the dermis. The scaffolds mimicking the dermis can be made by either natural or synthetic polymers (or a combination of both) and, regardless of their origin, they must have different characteristics: non-immunogenic; biocompatible; be able to resist the activity of proteolytic enzymes; stiff and flexible in order to withstand surgical procedures; be able to control the wound contracture; and to possess a degradation rate synchronous with the neo-dermis ingrowth and assembling. Furthermore, the scaffold should support epidermis attachment, maintenance and stratification, and it should promote the blood vessels’ influx when implanted [23,34,42]. Three-dimensional porous structures can be obtained by different fabrication techniques allowing the production of different 3D architectures. Starting from melt polymers or polymer solutions, the use of porogen agents, or phase separation techniques, allows the production of sponge-like structures with interconnected pores and porosity ranging from 50 to 500 μm [44]. When porogens are used, the final mechanical properties of the scaffold can be modulated by varying the polymer concentration, the volume fraction and the dimension of the porogens. Phase separation technique exploits the thermodynamic instability of either polymeric solutions or blends [42]. The instability can be induced physically (i.e., temperature) or chemically (i.e., introduction of a solvent/non-solvent agent). The thermodynamic instability induces a segregation process with the formation of a dispersed and a continuum phase. The dispersed phase forms globular structures and after its removal porosity is created. The parameters affecting the final properties of the scaffold (mechanical properties, porosity, pore diameter and pore interconnection) are the initial composition of the polymer solution and, in the case of thermally induced phase separation, the cooling/heating rate is used to induce the thermodynamic instability. Woven and non-woven assembly of nano-fibers using electrospinning allows the production of porous structures categorized as fibrillar scaffolds [30,45,46]. Nanofibrous materials can be also produced by means of different techniques such as self-assembly, phase separation, fiber bonding and electrospinning. This kind of structure is able to mimic better the fibrous nature of the natural extracellular matrix. In in vitro tissue engineering, such preformed structures (either porous or fibrous) are colonized by the cells after the preparation, since their fabrication techniques represent severe conditions for cell viability. This could represent a limitation because a homogenous cell seeding through the thickness of the scaffolds is difficult to achieve and sophisticated bioreactors need to be used [38,47,48,49]. Hydrogels represent another class of fibrous scaffolds. These are highly hydrated 3D structures obtained by physical, ionic or covalent cross-linking of different polymers of both natural or synthetic origins [49,50,51,52]. Three-dimensional hydrogels can be obtained by the assembly and crosslinking of a liquid monomeric phase. This represents an advantage because the monomeric solution can be mixed with the cell suspension. At the end of the gelling process, cells remain entrapped in the 3D structures obtaining a homogenous cellularization of the final 3D scaffold. Although the final properties can be modulated by different parameters (monomer concentration, temperature, pH, UV radiation, ionic strength) the presence of cells poses the same constraints on the control of the final mechanical properties. For instance, UV radiation, pH, temperature and other methods used to induce cross-link of polymeric networks may affect cell viability. Hydrogel are, in general, considered soft materials. Hydrogels composed of gelatin–chitosan hydrogels [53], fibronectin–hyaluronan, or dextran-based hydrogels, in combination with nano-fibrous poly lactic-co-glycolic acid (PLGA) scaffolds have been extensively used for wound healing and regeneration applications [54,55,56]. In particular, dextran-based hydrogels demonstrated efficient vascularization. Composite hydrogels formed by glycosaminoglycan (GAG)–collagen showed good wound healing in rabbit models [56]. Self-assembling peptide-based hydrogel scaffolds have been reported to reduce burn wound healing time and skin cell proliferation [56]. 

Protein-based naturals biomaterials. Biomaterials of protein nature can be realized using (but not limited to) collagen, gelatin, silk and fibrinogen. Collagen is the most abundant structural protein of the human dermis secreted by fibroblasts, and is responsible for tensile stiffness. Collagen in skin tissue engineering is used as both acellular scaffold or cell-populated scaffold [56]. Collagen for tissue engineering application is extracted from animals: bovine, ovine and avian are the mostly exploited sources. Examples of acellular/porous scaffolds are the commercially available dermis substitutes Alloderm^®^ or Integra^®^, while examples of cell-populated collagen hydrogels are represented by Apligraft^®^ and Transcyte^®^. In addition, collagen-based biomaterials have been processed in the form of membranes, sponges, composite sponges and electrospun biomaterials with nanometric features [56]. Gelatin is a protein obtained by collagen denaturation possessing higher advantages in terms of cell adhesion and inducing a reduced immunogenic response. In the treatment of wounds and burns, gelatin has been used as electrospun nanofibers [57], membranes [58] and gelatin sponges loaded with growth factors [59]. Silk fibroin in the form of sponges, nanofibers and porous films have shown decreased inflammatory response, and promising results in wound healing and skin regeneration has been reported [60]. 

Polysaccharide-based biomaterials. Polysaccharide-based biomaterials are mainly used in the form of hydrogels. Those mostly used in skin regeneration and wound healing are dextran [54], cellulose [26], chitosan [61], alginate [62] and hyaluronic acid (HA) [63,64,65]. Among these, HA has been extensively used in skin regeneration leading to the commercialization of different skin substitutes, such as Hyaff^®^, Laserskin^®^ and Hyalograft^®^.

Synthetic and composite biomaterials. Synthetic biomaterials comprise [30] the class of aliphatic polyesters, such as polylactic acid (PLA), polyglycolic (PGA) and polycaprolactone (PCL). They possess controllable mechanical stiffness and high process flexibility and are biocompatible and nontoxic. Moreover, PLA, PGA, PCL, and their blends and copolymers are FDA approved. An example of a commercially available skin substitute made using such materials is Dermagraft^®^. 

Decellularized matrices. To date, no biomaterials yet exist that are able to mimic the composition of the native extracellular matrix as a whole [16,17,18]. Decellularized matrices of allogenic or xenogeneic origin should bridge such a gap. This is very important in skin regeneration because the lack of a functional extracellular matrix is the main cause affecting the impaired wound healing process. Currently available decellularized biomaterials comprise decellularized mesothelium, intestine, amniotic membrane, dermis and skin flaps [9]. Allogenic dermis can be obtained by treating fresh dermis from a cadaver with Dispase–Triton X-100 or NaCl–sodium dodecyl sulfate (SDS). In this way, collagen bundles and basement membranes retain their structure. The abstained biomaterial is further lyophilized. An FDA-approved decellularized dermis obtained with such a technique is Alloderm^®^ [9]. Its use in combination with split-thickness autologous skin grafts allows complete cellularization of skin defects after 12 weeks post application when applied to full-thickness or partial-thickness burn wounds, thereby reducing subsequent scarring [65]. Other acellular dermal matrices are of porcine origin (e.g., Permacol^®^) and the decellularization process is similar to that of Alloderm^®^. Different techniques use a foaming process in order to destroy the cellular component, as well as any immunogenic agent. On the other hand, foaming compromises extracellular matrix structure and functions. In general, this kind of decellularized dermis is similar to porous scaffolds and is used for hemostatic applications. Other kinds of decellularized matrices are derived from the mesothelium, including peritoneum, pleura and pericardium [27,66,67]. Decellularized peritoneum is obtained using detergent agents and the processes are designed to maximize the preservation of the extracellular matrix architecture and composition. Because growth factors have a limited shelf life, such biomaterials are often combined with fibroblast growth factor (FGF) and epidermal growth factor (EGF) to promote wound repair. Decellularized mesothelium of porcine origin is used, for instance, in breast reconstruction. Bovine sources are another font of decellularized mesothelium showing a faster healing process than that observed for other decellularized dermis substitutes such as Alloderm^®^ [27]. The intestine is another source to obtain decellularized extracellular matrices. Interestingly, many cytokines and growth factors (FGF and TGF-β families) are retained after the decellularization process. Different applications have been reported comprising the reconstruction of cornea, urethra, vagina, and lung. In the case of dermis reconstruction, decellularized intestine and, in particular, the decellularized small intestine submucosa, is a very promising scaffold due to its capability in promoting angiogenesis. OaSIS^®^ and SurgySIS^®^ (Cook Surgical, Bloomington, IN, USA) are two decellularized matrices from small intestine submucosa [9,68]. The human amniotic membrane is rich in basement membrane and avascular stromal matrix. Decellularization can be obtained using ethylenediaminetetraacetic acid (EDTA) and aprotinin, SDS, DNAase and RNAase. Nevertheless, such a process induces the reduction of anti-inflammatory and anti-scarring components by reducing their superiority compared to other matrices. Clinical applications can be found in the reconstruction of the ocular surface, while different studies have been performed on skin reconstruction in nude mice models [27]. 

### 2.2. Tissue Engineering Strategies

#### 2.2.1. Traditional Tissue Engineering

Tissue engineering aims at producing functional and living human tissue in vitro that can be implanted to restore and to replace damaged tissues and organs [32], or can be used in vitro as living testing platforms [68]. The classical approach involves the extraction of cells from humans (primary cells or stem cells), their expansion and seeding in a biomaterial [32], followed by a dynamic culture to promote the neo tissue growth [10,38,47,49]. After cell expansion, the skin tissue engineering process involves at least three steps. (i) Fibroblasts are seeded in a biomaterial; this cellular construct acts as a dermis surrogate where the scaffold represents the temporary extracellular matrix of the fibroblast. (ii) After a variable culture period (two weeks–one month), to allow fibroblast attachment and production of endogenous extracellular matrix components, keratinocytes are seeded on the top of the engineered dermis and kept in submerged culture. (iii) After approximately two weeks, the culture conditions are switched from submerged to air liquid interface (ALI) in order to promote the stratification of the epidermis with the formation of the stratum corneum [2,69]. Such full-thickness engineered skin can be eventually implanted, but different requirements need to be satisfied [70]. The engineered skin must be safe for the patient because any cultured cell material possesses an associated risk of contamination, and viral or bacterial infection. Moreover, in the case of allogenic or xenogeneic cells and materials, there is also an associated risk of rejection. The tissue engineered skin should be effective in providing real benefit for the patient: it should attach correctly to the wound area, it should undergo normal healing limiting or discouraging the formation of a scar, and it should restore the correct pigmentation and barrier functions. Then, it should support the vascular network ingrowth [2,24] with subsequent development of other structures useful for the normal life of the patient: innervation should be promoted in order to restore the sensing properties [71] and the growth of both hair follicles and sweat glands [72]. Finally, tissue engineered skin should be cost-effective in order to achieve a concrete clinical uptake. One of the strengths of the tissue engineering approach is the possibility to have fine control over the properties of the final tissue. Indeed, by modulating the initial properties of the scaffold used to accommodate human fibroblasts and optimizing the culture conditions (culture media composition, mechanical stimulation, hydrodynamic stimulation) it is possible to obtain engineered skin with desired properties [10,38,47,48,49,70]. It has been demonstrated that uniaxial and biaxial stretch can induce the alignment of the de novo synthesized collagen network [73]. Furthermore, by engineering either the stiffness or the porosity of the scaffold, it is possible to control the assembly of the de novo synthesized extracellular matrix [74]. This allows, for instance, to match the final properties of the engineered skin with the properties of the patient’s skin in order to lower the structural and functional differences between the restored zone and the surrounding skin [13,21,43,75,76,77,78].

#### 2.2.2. Modular Tissue Engineering: Building a Tissue from the Bottom Up

**Figure 3:**
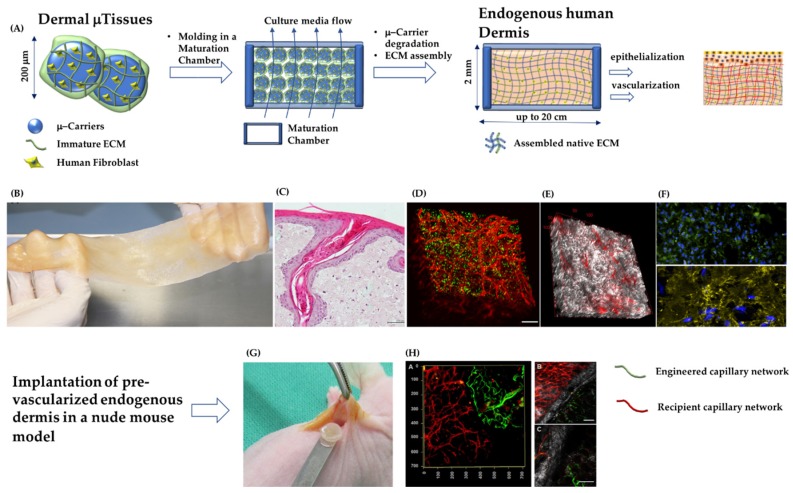
The main steps for the production of a DRT composed of fibroblast-assembled/pre-vascularized human dermis substitutes, and its morphological features before and after implantation in a nude mice model. (**A**) From left to right: production of Dermal-μTissues; their molding and assembly in a maturation chamber that is kept under dynamic culture conditions; formation of a continuum of fibroblasts embedded in their own dermal extracellular matrix; epithelization and vascularization of the endogenous human dermis. (**B**) Fabrication of large pieces of endogenous human dermis (major dimension 20 cm). (**C**) Histology of the endogenous human dermis supporting the differentiation of epidermis with the formation of spontaneous rete ridge profile. (**D**) Vascularized endogenous human dermis: cell nuclei in green and capillary network in red. (**E**) Vascularized endogenous human dermis: fibroblast-assembled collagen bundles observed under label-free multiphoton microscopy in gray; capillary network in red. (**F**) Top: fibroblast-assembled hyaluronic acid in green, cell nuclei in blue; Bottom: fibroblast-assembled elastin network in yellow, cell nuclei in blue. (**G**) Implantation of a piece of the pre-vascularized endogenous human dermis. (**H**) Connection between engineered capillary network (green) and recipient capillary network (red); fibroblast-assembled collagen in gray. Figure 3B, 3D, 3E, 3G, and 3H are from reference [34] “Mazio, C. et al. Pre-vascularized dermis model for fast and functional anastomosis with host vasculature. Biomat. 192, 159–170 (2019)”. Authors obtained permision from Elsevier: License Number 4681910194044.

Modular tissue engineering strategy applies the concept of tissue engineering but at a sub-millimeter scale. Cells are arranged in 3D architectures in the form of micromodules or micro tissues, or building blocks having at least one dimension ranging from 50 to 200 μm. Such micrometric tissues can be either scaffold-free or scaffold-based, and they act as building blocks for the fabrication of larger structures [78]. Scaffold-free microtissues can be obtained by organizing cells in sheets [79] (with a thickness ranging from 50 to 100 μm) or spheres [80,81] (diameters up to 200 μm). Scaffold-based microtissues are obtained by entrapping cells in micrometric scaffolds, such as porous microspheres [81], non-porous microspheres [82,83,84,85], non-spherical microparticles [78] or wires of hydrogels [84]. Sheets of human fibroblast are obtained by culturing fibroblasts in flat dishes and promoting the synthesis of the extracellular matrix. When the sheets achieve confluency, they are detached from the culture dishes and, by stacking different sheets of cells, a thicker tissue can be obtained [85]. When placed in close proximity, cell–cell contacts and extracellular matrix–extracellular matrix contacts lead to the formation of continuum structures made by fibroblasts embedded in their own extracellular matrix. One of the limitations of this technique is represented by the high cell density. This induces the formation of a dermis equivalent featuring a cell: ECM ratio higher than that found in the human dermis. The presence of high traction forces exerted by fibroblasts on the immature collagen fibers induces the formation of a highly packed ECM. Moreover, the over-expressed cell density increases the metabolic request. For these reasons, the cell-sheets are often characterized by a very low thickness and by the presence of a necrotic core. The detachment of the cell sheets from the culture plate and the subsequent stacking procedures represent other issues [86,87,88]. The fabrication of centimeter-sized tissues can be obtained by casting spherical microtissues in molds having any shape and dimensions [83]. Fibroblasts can be entrapped in both spherical and non-spherical hydrogels under continuous conditions using microfluidic devices [89,90]. Dermal microtissues obtained using this approach have been successfully used to build up large pieces of living dermis in vitro. In this direction, fibroblasts laden hydrogel has been used as a building block to fabricate a doll-shaped dermis equivalent [83]. In this study, the possibility of building up a centimeter-sized piece of dermis was demonstrated, but the final tissue underwent sever contracture. Since fibroblasts were imbedded in collagen hydrogel, the lack of a mature cell-synthesized extracellular matrix capable of withstanding the traction force of fibroblasts caused the shrinking of the final tissue. Finally, the presence of an exogenous collagen did not guarantee the complete replication of the native extracellular microenvironment [83]. To reduce the presence of exogenous matrices and promote the synthesis and the assembly of an endogenous dermal microenvironment, human fibroblasts have been seeded in porous gelatin microspheres kept in suspension cultures [89]. Under optimized culture conditions, fibroblasts were able to produce and assemble their extracellular matrix in the inner pores of the microspheres. The microspheres were designed in order to degrade during the extracellular matrix assembly process so the final microtissue was a sort of a sub-millimeter-sized “ball of human dermis”, named Dermal-μTissue (Figure 3), composed of fibroblasts and fibroblast-assembled-collagen, elastin and hyaluronic acid [83,91,92]. Dermal-μTissues, having an average diameter of about 200 μm, have been cast in centimeter-sized molds in order to promote biological sintering. The molds containing the Dermal-μTissues have been inserted in bioreactors working under engineered fluid dynamic regimes, which have been developed to improve mass transport during the assembly of the Dermal-μTissue [91,92]. In these works, shear stress and optimized fluid velocity fields were used to guide the correct assembly of the de novo synthetized ECM [92,93]. Moreover, the final dermis equivalent was completely formed by a fibroblast assembled extracellular matrix, leading to the fabrication of skin substitutes with superior functionalities compared to the engineered skin composed of exogenous ECM. For instance, when cultured in vitro in the presence of human keratinocytes and dorsal root ganglion cells, the first spontaneous formation of follicle-like structures in vitro [72] and functional innervation [35] were observed. Finally, tissue wire technology can be used to produce living fibers treated as textile and woven fibers [84]. These modular approaches lead to several advantages, such as fine control over the final architecture, control over the final shape, and control over the spatial organization of engineered biologic structures [79,80,81,82,83,84,85,86,87,92,93]. 

##### Three-Dimensional Bio-Printing

Three-dimensional bio-printing [94] techniques are used to achieve correct positioning of different cell types. The aim is the fabrication of intricate biologic architectures with high spatial resolution in a standardized manner [82,95,96,97]. Moreover, the obtainment of a full-thickness human skin equivalent takes at least four weeks, representing an issue in the case of production of autologous skin engineering grafts that should be implanted in a shorter time frame. The positioning of different cell strata in their final configuration could reduce the time required for the development of the full-thickness graft. Other advantages of 3D printing techniques are represented by the possibility of printing a vascular network and its insertion in its final configuration in important adnexal structures such as the hair follicle precursors [97,98,99,100]. This should represent a plus in the case of deep skin damage where neither innervation nor hair follicle development during the healing process has been observed [20]. Finally, the use of 3D printing techniques aims at reducing the batch-to-batch variability by providing a standardized and controlled process. By precisely locating different matrix materials, growth factors, and different cells in a layer-by-layer assembly, functional living skin tissues would be fabricated possessing designed and personalized structures with neither size nor shape limitations, in a high throughput, and in a highly reproducible manner. In general, the equipment used to print skin tissues consists of independently controlled cell-dispensing channels. Electromechanical valves operate the dispenser, which is positioned on a three-axis robotic stage possessing high spatial resolution (below 50 μm). The dispenser can deliver pre-hydrogel solutions containing cells. Once delivered, the hydrogel solution solidifies via chemical or physical routes. According to this strategy, fibroblasts embedded in rat tail collagen have been printed layer-by-layer, forming a dermis surrogate upon which keratinocytes have been deposited in order to form the epidermal layer [101]. In this study, it was demonstrated that a 3D dermis can be formed in an automated and controlled fashion by printing nanoliter droplets of collagen containing living cells [95,99]. A printed skin equivalent can be obtained using fibrin hydrogels as bio-ink containing cells [95,100]. It was demonstrated that a dermis equivalent as large as 100 cm^2^ could be obtained in less than 35 minutes. After this time, a layer of keratinocytes was printed on the top of the dermis equivalent and, after confluency was obtained (24 h), the bilayer engineered skin was implanted in immunodeficient mouse. After implantation, the dermis was able to integrate with the recipient tissue and the epidermis was able to differentiate until forming the stratum corneum. Nevertheless, the dermal–epidermal interface did not present the physiological rete ridge profile. Finally, techniques for the printing of a microvasculature network have been assessed. Endothelial cells can be inserted in fibrinogen solution and printed in a gelling bath according to a prescribed 3D architecture. Together with the vascular network, a hydrogel solution containing fibroblasts can be printed by means of an independent dispenser in order to obtain a more complex dermis formed by a connective tissue equivalent containing a 3D designed microvasculature [102]. 

## 3. Commercially Available Skin Substitutes

### 3.1. Acellular Dermal Substitutes

The most used commercially available acellular dermal substitutes (Table 1) are composed of natural extracellular matrix components and can be divided in two categories: decellularized extracellular matrices and reconstructed extracellular matrices. The first category is formed by natural connective tissues (dermis, mesothelium, intestine) deprived of any cellular components and allergenic/immunogenic agents.

The most used commercially available decellularized matrices (Table 1) for the treatment of deep wounds after burns and trauma, or for reconstruction and treatment of diabetic/venous/pressure ulcers are (but not limited to): Alloderm^®^, Dermacell^®^, Dermamatrix^®^, SureDerm^®^, OASIS^®^, Permacoll^®^ and EZ-DERM^®^. Alloderm^®^, Dermacell^®^, Dermamatrix^®^ and SureDerm^®^, are decellularized cadaveric dermis, non-cross-linked, that can be incorporated into the wound bed [4,6,7,8,9,40,42,103,104]. In general, such systems retain the basement membrane after the decellularization process but lack an epidermal layer. The acellular matrix provides a good natural 3D environment for fibroblasts and endothelial cells influx in order to promote the formation of a new extracellular matrix and vascular network. OASIS^®^, Permacoll^®^ and EZ-DERM^®^ are decellularized matrices of porcine origins. OASIS^®^ is obtained using similar processing methods to those of human derived matrices but start from porcine small intestine submucosa. Permacoll^®^ and EZ-DERM^®^ are decellularized porcine dermis that are further cross-linked. Alloderm^®^ was approved and considered as banked human tissue by the FDA; it has been used to treat burns since 1992 and has also been used to treat severe soft tissue defects [105]. This product has been shown to have good graft take rates and to reduce subsequent scarring of full-thickness wounds, even though the graft take of split-skin grafts in a one-step procedure is low. Alloderm^®^ is considered medically necessary in post-mastectomy breast reconstructive surgery for at least one of the following indications: there is insufficient tissue expander or implant coverage by the pectoralis major muscle and additional coverage is required; there are thin post-mastectomy skin flaps that are at risk of dehiscence or necrosis; or the infra-mammary fold and lateral mammary folds have been undermined during mastectomy and re-establishment of these landmarks is needed [39]. By retrieving information form the websites of Dermacell^®^ and Dermamatrix^®^, it is possible to note that they are intended for soft tissue reconstruction (face defects, nasal reconstruction, abdomen, etc.) and for breast reconstruction. Moreover, different clinical trials involving Dermacell^®^ and Dermamatrix^®^ can be retrieved by consulting the database of clinicaltrials.gov. SureDerm^®^ is indicated by the manufacturer as suitable for gingival and root reconstruction. EZ-DERM^®^ is a porcine-derived xenograft in which the collagen has been chemically cross-linked with aldehyde in order to provide strength and durability [106]; it has FDA 510(k) approval for the treatment of partial-thickness burns and venous, diabetic, and pressure ulcers. In a randomized study involving 157 patients affected by partial-thickness burns, it was found to have satisfactory results: non-correct positioning = 1.5%, infection = 3.0%, incomplete epithelialization at time of separation = 2.2%, need for additional excision and grafting = 4.5%, and hypertrophic scaring = 3.3% [106]. OASIS^®^ Wound Matrix and OASIS^®^ Ultra Tri-Layer Matrix are considered medically necessary for treatment of chronic, noninfected, partial- or full-thickness lower-extremity vascular ulcers, which have not adequately responded following a one-month period of conventional ulcer therapy. They are regulated by the FDA as a Class II (moderate risk) device and received FDA 510(k) approval (K061711), on 19 July, 2006 [39]. OASIS^®^ Wound Matrix was subjected to a randomized controlled study in 120 patients with chronic venous leg ulcers. Significantly more wounds (55% vs. 34%) were observed to be healed in comparison with conventional therapy [67]. 

Using other routes, the production of acellular dermis is obtained in vitro using the main constituents of the connective tissues (collagen, elastin, glycosaminoglycan, etc.) which are extracted from animals and then reconstructed. Once extracted and purified, the extracellular matrix components can be eventually combined together and processed to form 3D porous structures according to the techniques discussed in Section 2.1 (e.g., cross-linking, lyophilization and electrospinning). In the class of reconstructed extracellular matrices (Table 1), we find: Integra^®^, Biobrane^®^, Matriderm^®^ and Hyalomatrix^®^. Integra^®^ Dermal Regeneration Template consists of a bi-layered extracellular matrix of fibers of cross-linked bovine collagen and chondroitin-6-sulfate (a component of cartilage) with a silicone membrane as transient epithelium. Once the neodermis is formed, the disposable silicone sheet is removed, and an ultrathin autograft is placed over the neodermis [9,20,26]. Integra^®^ Dermal Regeneration Template is considered medically necessary in the post-excisional treatment of severe burns when autografting is not feasible. It has an FDA PMA for treatment of life-threatening, full-thickness or deep partial-thickness thermal injuries where sufficient autograft is not available at the time of excision or not desirable due to the physiologic condition of the individual [39]. This product also has an FDA PMA for repair scar contractures. An issue affecting this kind of skin substitute is the time required for neovascularization. Indeed, when the dermis bed is not well vascularized, or it takes a long time to achieve vascularization, the take of the STSG is compromised. Matriderm^®^ becomes vascularized faster than Integra^®^, supporting the take of a split-skin graft in a one-step procedure. This is due to the presence of elastin in the Matriderm^®^ model, which is able to attract more vascular cells than the chondroitin-6-sulphate present in Integra^®^. Biobrane^®^ is an acellular dermal matrix composed of bovine type 1 collagen, silicone and nylon, and mechanically bonded to a flexible knitted nylon fabric. The semipermeable membrane is comparable to human epidermis and controls the loss of water vapor, allows for drainage of exudates, and provides permeability to topical antibiotics. The nylon/silicone membrane provides a flexible adherent covering for the wound surface [2,6,9,10,31,39,41,57,61,67,76,99,107,108]. Biobrane^®^ holds an FDA 510(k) approval for the treatment of clean partial-thickness burn wounds and donor site wounds. It is considered medically necessary [39] for the treatment of burn wounds when all of the following criteria are met: the treatment is specific to non-infected partial-thickness burn wounds and donor site wounds; excision of the burn wound is complete (e.g., nonviable tissue are removed) and homeostasis achieved; sufficient autograft tissue is not available at the time of excision; and autograft is not desirable due to the individual’s physiologic condition (e.g., individual has multisystem injuries such that creating new wounds may cause undue stress). It has been shown to be as effective as frozen human allografts. Furthermore, when used on excised full-thickness burns, it reduces hospitalization time in the case of pediatric patients with second-degree burn injuries. Nylon present in Biobrane^®^ is not incorporated, making such an acellular matrix a wound dressing rather than a skin substitute. Hyalomatrix^®^ [2,6,9,42,107,109,110,111] is a bilayer, esterified hyaluronic acid (HYAFF^®^) matrix with an outer silicone membrane. The connective-mimicking layer HYAFF^®^ is a long-acting derivative of hyaluronic acid providing a microenvironment suitable for optimal tissue repair and accelerated wound healing. Specifically intended for the treatment of deep burns and full-thickness wounds, it also provides a wound preparation support for the implantation of autologous skin grafts.

### 3.2. Cellularized Dermal Substitutes

Cellularized skin substitutes can be divided into three categories: (1) Epithelial sheets: formed by epithelial cells embedded in or seeded on polymeric membranes. Such engineered epithelial tissues will not be discussed in this review because the scope of the survey is to elucidate the role of dermis regeneration during the closure of deep wounds. (2) Dermis equivalents: composed of 3D porous matrices or hydrogels containing fibroblasts. (3) Full-thickness (or composite) skin equivalents: composed of a dermis equivalent and epidermis.

In the class of cellularized dermis (Table 2) we find (but are not limited to): Dermagraft^®^, TransCyte^®^, and Hyalograft3D^®^. Dermagraft^®^ is classified by the FDA as an interactive wound and burn dressing approved under the PMA process as a class III, high-risk device, and requires clinical data to support their claims for use: treatment of full-thickness diabetic foot ulcers of greater than six weeks duration that extend through the dermis, but without tendon, muscle, joint capsule, or bone exposure. It is a living dermal replacement composed of a bio-absorbable PLGA mesh seeded with cryopreserved neonatal allogeneic foreskin fibroblasts. Dermagraft^®^ is considered medically necessary when used for at least one of the following indications: the treatment of full-thickness diabetic foot ulcers of greater than six weeks duration that have not adequately responded to standard therapy, that extend through the dermis, but without tendon, muscle, joint capsule or bone exposure; or when used on wounds with dystrophic epidermolysis bullosa. It is advised that this material should be used in patients that have adequate blood supply [39]. This dermal substitute appears to produce results as good as allografts with regard to wound infection, wound exudate, wound-healing time, wound closure and graft take, and is more readily removed than allograft, with significantly higher levels of patient satisfaction [111]. The advantages of this skin substitute include good resistance to tearing, ease of handling and lack of rejection [112]. TransCyte^®^ is a nylon mesh coated with bovine collagen and seeded with allogenic neonatal human foreskin fibroblasts which proliferate and synthesize growth factors and extracellular matrix components. It was shown that the presence of a cell-assembled ECM was able to hasten the re-epithelialization process of partial-thickness burns [113]. Furthermore, a multicenter randomized clinical study showed it to be even superior to frozen human cadaver allograft for the temporary closure of excised burn wounds [114].

TransCyte^®^ received an FDA PMA as a temporary wound covering for surgically excised full-thickness and deep partial-thickness burn wounds (detailed reports can be retrieved on the web site of the FDA). It is considered medically necessary for the following uses: temporary wound covering to treat surgically excised full-thickness (third-degree) and deep partial-thickness (second-degree) thermal burn wounds in persons who require such a covering before autograft placement; and the treatment of mid-dermal to indeterminate depth burn wounds that typically require debridement, and that may be expected to heal without autografting [39].

Hyalograft3D (FDA 510(k) approval) comprises esterified hyaluronic acid fibers seeded with autologous fibroblasts and covered by a silicone membrane. Its use in diabetic ulcer therapy has been reported, showing significant improvement of the wound closure compared to other devices [115]. Apligraft^®^ consists of neonatal fibroblasts seeded onto a bovine type I collagen gel and neonatal keratinocytes cultured on top of this dermal layer [116,117,118]. It gained an FDA PMA based on its efficacy with venous ulcers. Apligraf^®^ also has an FDA PMA for use in the treatment of diabetic foot ulcers.

In the case of venous insufficiency, it is considered medically necessary for at least one of the following indications: chronic, non-infected, partial- or full-thickness ulcers due to venous insufficiency; standard therapeutic compression also in use; and at least one month of conventional ulcer therapy (such as standard dressing changes, and standard therapeutic compression) has been ineffective [39]. In the case of diabetic foot ulcers, it is considered medically necessary for at least one of the following indications: full-thickness neuropathic diabetic foot ulcers that extend through the dermis but without tendon, muscle, joint capsule, or bone exposure; and at least four weeks of conventional ulcer therapy (such as surgical debridement, complete off-loading and standard dressing changes) has been ineffective. It has been reported that adding Apligraf^®^ to compression therapy for chronic venous ulcers doubled the number of healed wounds at six months. Furthermore, in chronic diabetic foot ulcers, 56% of patients in the Apligraf^®^ group had reached complete healing by 12 weeks compared with only 38% in the control group with moist gauze dressing treatment [118]. Use of Apligraft^®^ in other skin defects, such as donor-site wounds and epidermolysis bullosa [119], has been reported. Disadvantages are its uneven pigmentation and contracture, short shelf life of five days, fragility, the risk of disease transfer due to its allogeneic constituents, and the high cost [4]. Tissuetech^®^ is composed of hyaluronic acid-based matrix seeded with fibroblasts with autologous keratinocytes on top. It was shown to be effective in the treatment of the lower limbs. Furthermore, in 401 diabetic ulcers, 70.3% treated with Tissuetech^®^ healed within less than one year [2,42,107]. OrCel^®^ is a bi-layered skin substitute formed by human derma fibroblasts entrapped in a bovine collagen sponge, with human keratinocytes on top.

OrCel^®^ has received an FDA PMA for the treatment of fresh, clean split-thickness donor sites associated with mitten-hand deformities in individuals who have recessive dystrophic epidermolysis bullosa. It is considered medically necessary for the following indications: epidermolysis bullosa in children after reconstructive surgery; and full-thickness (third-degree) or partial-thickness (second-degree burns) [3,39,41,116,120,121]. Once placed in the defect, OrCel^®^ dissolves and is replaced by the patient’s own skin. 

### 3.3. Clinical Effectiveness of Skin Substitutes

#### 3.3.1. Effectiveness of Acellular Dermal Regeneration Templates

Dermal substitutes must guarantee correct regeneration of the dermis compartment [13]. After implantation, a dermal substitute has to allow a fast recruitment of endothelial cells in order to guarantee the correct take of a thin STSG. As a long-term aim, the skin substitute should guide a correct regeneration avoiding the formation of scar tissue. It has been recognized that an ideal dermal substitute must satisfy the following requirements [38]:

- avoid the immune response, inflammation and any kind of rejection;
- protect the wounds from infection and loss of fluid;
- easy to handle and flexible, but stiff enough to withstand surgical procedures;
- enable the influx of cells (fibroblasts and endothelial cells) that will build the neodermis;
- stable enough to guarantee the correct neo-synthesis and assembly of immature extracellular matrix; on the other hand, its rate of degradation should be synchronous with the rate of formation of the neo-tissue;
- enable a correct vascularization in less than 14 days post implantation in order to increase the probability of the take of the STSG;
- be able to guide the regeneration avoiding the formation of scar tissue.

To date it is difficult to make a comparison between the efficacy of the different commercially available dermal substitutes because of the lack of long-term follow-up studies in which different dermal substitutes have been studied in parallel. Even where different clinical studies exist for each of the skin substitutes, it is difficult to make an objective comparison due to different factors. Notably, different studies use different methods for the evaluation of the success of the healing. Indeed, different scales exist to evaluate the features of the scar after implantation: Vancouver Scar Scale, Hamilton Burn Scar Rating, Patient and Observer Scar Assessment Scale, Manchester Scar Scale and Visual Analog Scale [121,122,123,124]. Moreover, the studies are conducted starting from different type of wounds (burns, ulcers, trauma, etc.). Finally, the experience of the surgeon plays a crucial role in the success of the take of both dermal regeneration template and STSG. This is highlighted by analyzing the results on the take of grafts coming from multicenter and single center studies: the former possesses higher degrees of uncertainty than the latter [124]. 

Nevertheless, it is possible to extrapolate some general indications showing the benefits and downsides of the different dermal substitutes. By focusing attention on partial- to full-thickness wounds, the gold standard treatment is the application of a partial- or full-thickness STSG. The application of an STSG is an autografting procedure and possesses two main issues: (i) the use of an STSG is an invasive technique because after the removal of large parts of healthy tissues, other damage is induced; and (ii) if the total burn surface is higher than 25% of the total body area, the donor site cannot provide enough tissue to cover the wounded area. For this reason, the majority of the clinical trials have been conducted by comparing the efficacy of a dermis substitute to the efficacy of the gold standard treatment. Among the acellular dermal substitutes, Integra^®^, Matriderm^®^ and Alloderm^®^ have been subjected to a systematic review [38] thanks to the availability of long-term follow-up studies starting from similar wounds (partial- or full-thickness wounds due to thermal burns). The comparison between the dermal substitutes and the control groups has been performed by analyzing primary outcomes (graft take, wound infection, scar quality) and secondary outcomes (donor site morbidity, convalescence of the patient, need to re-graft). Compared with an STSG, only Integra^®^ showed a significantly lower take (*p* < 0.001). Concerning the infection rate, a value of 85% of infection in patients having a total burn surface of 45% and treated with Integra^®^ has been reported [125,126]. However, other studies reported no difference in infection percentage between Integra^®^ and the control group [26,126,127]. A 6% rate of infection has been detected in a study where Alloderm^®^ was used [127]. Matriderm^®^ did not reveal any difference of infection rate compared with the controls [128]. Concerning scar quality, data only showed better scar quality for Integra^®^ compared to that of an STSG. For all other models, no significant differences were detected in scar quality compared with the control groups. A relevant increase in donor site healing (lower donor site morbidity) has been detected in all studies [38]. This is because by using a dermal substitute to fill the wound bed, the thickness of the STSG can be reduced to 0.006 of an inch, compared to 0.01 of an inch for gold standard treatments. 

With the exception of Integra^®^, few data concerning the mortality and length of stay of patients have been found [38]. No significant differences in rates of re-graft were reported between the dermal substitutes and control groups. Other studies have compared five dermal substitutes (involving Integra^®^, Matriderm^®^ and Hyalomatrix^®^) in a preclinical pig model [110]. No difference was found at the end of the survey (six months after implantation) in terms of scar quality and healing properties. A difference was found in the evolution of the healing process. Integra^®^ took more time for fibroblast and blood vessel influx and was still present, as a fragmented matrix, at the end of the survey. Hyalomatrix^®^ showed a lower degradation rate than Matriderm^®^, but both had completely disappeared at the end of the survey. The quality of the scar assessed with the Vancouver Scar Scale was similar. A long-term follow-up revealed both histological and clinical outcomes in a randomized study using Integra^®^ and Nevelia^®^ [103]. Nevelia^®^ is a porous degradable matrix of about 2 mm thickness made of stabilized native collagen type 1 from calf hides and a silicone sheet of about 200 μm thickness mechanically reinforced with a polyester fabric, and does not contain any chondroitin-6-sulphate GAG. Collagen is purified from calf hides from animals younger than nine months sourced from safe countries. Collagen is then cross-linked with a very low percentage of glutaraldehyde. Clinical results were evaluated through the healing time, Manchester Scar Scale (MSS) and Visual Analog Scale (VAS) up to three years. The differences in healing time between groups, pain and self-estimation was not statistically significant up to a one-year follow-up. Nevelia^®^ showed early regenerative properties in terms of epidermal proliferation and dermal renewal at three weeks, compared with Integra^®^. Furthermore, Nevelia^®^ showed a more evident angiogenesis vs. Integra^®^, evaluated as α-SMA immunohistochemistry. Differences in the MSS score were statistically significant at three years follow up in favor of Nevelia^®^ group (*p* = 0.001), together with clinical outcomes. Histological and immunohistochemistry data showed that Nevelia^®^ allows faster neo-angiogenesis and tissue regeneration with neo-formed tissue architecture closer to the physiology of the skin. This data confirms the importance of both vascularization and neodermis influx within the dermal substitute. Indeed, it is well accepted that the faster the vascularization, the higher the take of the STSG. Integra^®^ contains a fraction of chondroitin-6-sulphate GAG that had two side effects: firstly, such a kind of GAG slows the influx of endothelial cells; secondly, the presence of such a high hydrophilic component may retain lesion inflammatory exudates. Furthermore, the high degree of cross-linking in Integra^®^ masks both the adhesion site for fibroblasts and the proteolytic degradation sites, hampering the recipient cells’ influx and the degradation of such exogenous ECM. Finally, it seems that rapid vascularization and presence of endogenous dermal cells (fibroblast and vascular network) may play a crucial role in the effectiveness of the dermal substitute. 

#### 3.3.2. Effectiveness of Cellularized Skin Substitutes

The introduction of cellularized skin substitutes in the treatment of full-thickness wounds has been necessary in order to overcome some limitations of the acellular dermis. In particular, the presence of dermic cell lines (fibroblast or endothelial cells) serves to reduce the time for neodermis influx after implant. By using acellular dermal substitutes, surgeons have to wait at least four weeks for the implantation of the STSG in order to allow fibroblast and endothelial cells’ influx. Without such cellular components, the take of the STSG is not possible. Moreover, the larger the delay before the STSG is implanted, the higher the probability of infection. Thus, the success of the regeneration process is strictly related to both “colonization” time and the quality of the neodermis formed in the porosity of the acellular dermis. A cell populated scaffold introduced in the wound bed aims at reducing such colonization time as a primary task. We can highlight additional requirements that engineered skin substitutes have to possess compared with acellular substitutes:

- Safety concerns: any cultured cell material carries the risk of transmitting viral or bacterial infection, and some support materials (such as bovine collagen and murine feeder cells) may also have a disease risk.
- Clinical efficacy: since the biological risk is higher, the benefits in terms of quality of the healed tissue must be significantly superior, and not only equal, to conventional therapies.
- Convenience: in general, the cost of tissue engineered products is at least ten-fold higher compared to that of non-cellularized materials; in order to achieve clinical uptake, the benefits must include the reduction of hospitalization time, surgical operations after the implants, pain and the associated cost of the treatment.

To date, the use of tissue engineered products for the treatment of burns, although it has received broad scientific success, seems to be not convenient in terms of commercial outcomes. For this reason, many companies have focused their attention on the commercialization of engineered tissues for the treatment of chronic diseases: venous valve insufficiency, arterial diseases, diabetes, vasculitis, skin malignancies and blistering diseases. Regardless of the origin of the wound, the major aim is to reestablish the physiological conditions of the dermis layer. The cells that act to maintain the dermis are the fibroblasts [108], which synthesize and assemble collagen, elastin, and proteoglycans. When inflammation occurs, fibroblasts migrate to the wound site, attracted by bFGF and TGF-β secreted by inflammatory cells and platelets, where the fibroblasts are stimulated to replicate, migrate into the wound, and secrete IGF-1, bFGF, TGF-β, platelet-derived growth factor, and KGF, enabling fibroblast–keratinocyte interaction. When the wound is chronic, the continuous inflammation state induces a premature and stress-induced cellular senescence of the fibroblasts. Moreover, a decreased proliferative potential, impaired capacity to react to growth factors and abnormal protein production is observed. When the percentage of senescent cells in the defect is greater than 15%, wounds are described as hard to heal [14,15,129,130]. The use of Dermagraft^®^ for the treatment of non-healing ulcers has been demonstrated in different clinical studies [130,131,132]. Summarizing the results, it is possible to establish that the percentage of healing using Dermagraft^®^ ranged from 50% to 71.4%. The complete closure of the wound can be obtained only for ulcers with 12 months of duration or less.

The most popular bi-layered skin substitute (containing fibroblasts and keratinocytes) is Apligraf^®^, which has been studied since 1999. It has been demonstrated that use of such a bi-layered engineered skin is an effective treatment for ulcers of greater than one-year duration, with a percentage of wound closure of about 50%. Moreover, the number of osteomyelitis and lower-limb amputations were less frequent in the Apligraf^®^ group. The advantage of dermal–epidermal substitutes is the presence of the living epidermal layer that avoids the use of gauzes and two-step procedures. This reduces the risk of infections and improves the healing process due to the presence of fibroblast–keratinocyte cross-talk, which plays a crucial role in the healing process of deep wounds. One of the limitations of the aforementioned categories of skin grafts is the presence of allogenic cell lines, which contain an associated biological risk. This limitation is being overcame by developing models containing autologous cell lines. Two models that use autologous fibroblasts and keratinocytes are denovoDerm^®^ and denovoSkin^®^, which are under clinical trials at University Children’s Hospital of Zurich [71,133,134,135]. Finally, to deeply investigate on both FDA status and the effectiveness of current skin substitutes the reader can refer to “FDA” and “clinicaltrial” databases by searching for the desired skin model [136,137].

## 4. Advanced Bioengineered Skin Equivalents: A Future Perspective

### 4.1. Pre–Vascularization of Dermis Substitutes

The treatment and the evolution of deep wounds due to thermal burns is schematized in Figure 2A-D. After the debridement of the wound, the bed is filled with a DRT supporting an artificial layer of silicone-based epidermis. After a period of four weeks, the epidermal layer is detached and an autologous STSG is applied. In a clinical study that used Integra^®^ as the DRT, 20 patients presenting deep wounds were treated using the procedure described in Figure 2A-D. The evolution of the wound was analyzed by means of histology, immunocytochemistry and the Vancouver Scar Scale [20]. It was observed that the vascularization of the DRT played a crucial role in the take of the STSG. For instance, if the STSG was applied after two or three weeks, the take rate was very low. On the contrary, if the STSG was applied after the fourth week, the take increased up to 95%. Histological and immunostaining analyses demonstrated that at two weeks the vascularization of DRT was poor but increased four weeks after implantation. These data suggest that vascularization of the DRT and the take of the STSG are strictly related [20]. 

Other relevant findings concern the evolution of the dermis compartment over the time. Weekly histological investigation revealed that influx of exudates and host fibroblasts occurred during weeks one–two. At three weeks, the influx of endothelial cells and the synthesis of immature extracellular matrix components by fibroblasts began. During week four, the formation of a capillary network (Figure 2E) was observed. After the application of the STSG, the wound continued its evolution: at week six a well-organized capillary network was observed, but the dermis–epidermis interface presented no rete ridge profile; at month three, a layer of endogenous collagen network was observed underneath the STSG; after two months, the wound was completely repaired but the neo-tissue was different from the healthy skin. Finally, the complete substitution of the initial DRT with the neodermis occurred at two years post implantation. Even though the patients recovered partial mobility of the damaged parts, it was observed that the repaired zone showed an impaired pigmentation, the mechanical properties between healthy and repaired sites were different, and the organization of the collagen network of the neodermis was different than that of the collagen in the healthy dermis. Finally, neither elastin nor adnexa were present, and differentiation of fibroblasts in myofibroblasts was observed. On the basis of such findings two main issues affecting the DRT emerge: (i) the lack of vascularization [22,23]; and (ii) the limited capability in inducing regeneration instead of repairing processes [135]. The take of the STSG has huge implications related to the repairing process, patient mortality, and morbidity and healthcare costs. Indeed, a low take percentage increases the number of re-grafts and the risk of infection by causing either the death of the patient or an increase of hospitalization time in case of morbidity. To increase the take of STSGs, new emerging strategies involve the use of pre-vascularized DRTs [22,23,33,34,36]. By seeding a DRT with adipose tissue-derived microvasculature fragments, a faster vascularization after implants was observed [138]. Complete reperfusion of the DRT occurred at day six. The percentage of the take was high if the STSG was applied just after day six, indicating that reperfusion rather than simple vascularization played a crucial role in the take. These data suggest that pre-vascularization of the DRT can contribute to shortening the timeframe needed for the application of an STSG. On the other hand, a one-step surgery, which may decrease the number of surgical operations, cannot be performed yet. To do this, not only vascularization, but also fast reperfusion should be promoted. 

### 4.2. Engineered Skin Composed of Fibroblast-Assembled Extracellular Matrix

The lack of vascularization at the moment of implantation has been recognized as the main issue affecting the take of the STSG. No studies have been performed yet on the role that the extracellular matrix comprising the DRT may play on both vascularization and longtime dermal remodeling [17]. The dermis compartment of the totality of the skin substitutes (either cellularized or acellular) are composed by exogenous extracellular materials, i.e., not assembled by the fibroblasts of the patient. This should represent the limitation of the currently available tissue engineering skins. Indeed, exogenous matrices, even though of natural origins, cannot fully replicate the complexity of the living dermis. This may ultimately compromise the repository and regulatory role that the native cell-assembled extracellular matrix plays [16,17,18]. Such a mismatch between an exogenous material and the living dermis may be responsible for the impaired repair process at both cellular and extracellular levels. Firstly, because the repository and regulatory role of the native ECM is depressed, the growth factors secreted by fibroblasts are not correctly presented to other cell types (e.g., keratinocytes and endothelial cells) neither in space nor time, generating a possible “mistake” in cell–cell signaling [16,17]. This could explain both the delay in the vascularization time and the delayed formation of the rete ridge profile at dermal epidermal interfaces [20,26,72]. As confirmation of this, in vitro tissue engineered skin made by exogenous natural hydrogels (i.e., collagen, fibrin, etc.) presents a flat dermal–epidermal interface. On the contrary, if epithelial cells are grown on a fibroblast-assembled ECM, it is possible to observe a rete ridge profile with spontaneous formation of epithelial invagination and follicular-like structures (Figure 3C) [72], which are typical of the physiologic dermal–epidermal cross-talk mediated by the extracellular matrix [72]. The lack of endogenous ECM-mediated signaling may also explain the absence of both cutaneous adnexa and nerve endings in repaired deep wounds [20,26,129]. Secondly, when fibroblasts colonize the inner porosity of the DRT, they produce an immature extracellular matrix with a degree of assembly much lower than the degree of assembly of the surrounding healthy dermis. Such an immature protein network is not able to withstand the traction forces of the fibroblasts [74,110], generating a different architecture of the collagen fibers in the wound compared to the healthy dermis [15,19,21,77]. Macroscopically, these phenomena generate a portion of the cutis possessing different mechanical properties, different pigmentation, absence of sensing properties and high contracture, provoking both severe functional and aesthetic concerns. 

To overcome such limitations, a tissue engineering strategy to produce a human dermis substitute composed of a fibroblast-assembled extracellular matrix has been developed [73,75]. The innovative idea of such strategy is to let human fibroblasts producing their own ECM in vitro. This process provides the possibility of modulating the properties of the cell-synthesized ECM, in order to obtain a final dermis having both composition and assembly degree of the collagen network relatively similar to those present in vivo. Moreover, no exogenous materials are present. This bottom-up tissue engineering strategy starts with the fabrication of dermal building blocks obtained [81] by seeding human fibroblasts in porous gelatin microspheres (Figure 3A). It has been demonstrated that by optimizing the culture conditions, the fibroblasts can produce their own extracellular matrix. Such building blocks, named Dermal-μTissues, were subsequently molded and packed in maturation chambers where both cell–cell and ECM–ECM interactions took place, leading to the formation of a continuum, up to 2 mm thick, made of an endogenous dermis containing fibroblasts and gelatin microspheres. By modulating the stiffness and the degradation rate of the gelatin microspheres and by engineering the dynamic culture conditions (Figure 3), it was possible to obtain fine control over the maturation status and assembly of both collagen and elastin networks [74,91]. During the duration of the process (approximately five weeks), gelatin microspheres were degraded by protease digestion and the final tissue, named EndoDermis, was completely made up of fibroblasts embedded in their own extracellular matrix (Figure 3A). Interestingly, the collagen network was characterized by a stiffness and degree of assembly similar to that featuring the human skin. In the ECM elastin, hyaluronic acid, fibronectin and elastin were also present (Figure 3B-F). In order to produce a pre-vascularized endogenous human dermis model, human umbilical vein endothelial cells (HUVECs) were seeded on the EndoDermis and it was allowed to form an interconnected capillary network [34] that occurred within three weeks (Figure 3D, E). At the best of our knowledge, other than a capillary network, such an engineered DRT is the first model completely formed by a fibroblast-assembled extracellular matrix [34]. After subcutaneous implant in a nude mouse model, fibroblasts and their own ECM (the neodermis) were already present and well-assembled. Thus, no additional time is required for fibroblast influx and neodermis formation. The only phenomenon required is the anastomosis and perfusion of the engineered capillary network. This was shown to occur within seven days of implantation (Figure 3H). Although further investigations are currently being conducted of a more representative wound model, such data are encouraging. In addition to vascularization, which has been recognized as a critical issue [22,23,25,33,34] affecting the effectiveness of a DRT, the described tissue engineered strategy allows the fabrication of a DRT composed of a native extracellular matrix starting from a small number of fibroblasts derived from the patient. In this way, the risks associated with the allogenic nature of the cells and the impaired ECM assembly during wound closure, can be drastically reduced. According to this idea, the formation of severe scars can be reduced. 

## 5. Discussion and Conclusions

Dermal regeneration templates and tissue engineered skin [40] has been reviewed in the light of their effectiveness in guiding the closure of deep wounds toward a regeneration process rather than a repair process [38]. By analyzing the literature, no strategies are currently available that are able to completely restore the whole functionality of the reconstructed part, including pigmentation, mechanical properties, adnexal structures and sensing properties. In other words, the formation of severe scarring still represents a concern in the field of skin reconstruction. Many advances have been made to limit the use of thick STSGs. Indeed, using a last generation DRT, the thickness of STSGs can be reduced to 0.006 of an inch, compared to 0.01 of an inch for gold standard treatments. Furthermore, the take of STSGs has been improved by promoting the vascularization of the DRT [22,23,25]. It has been observed that a DRT composed of non-cross-linked matrices promotes the invasion of fibroblasts and endothelial cells, increasing the take of the STSG compared to the case of cross-linked matrices [9]. On the other hand, cross-linked matrices are able to better withstand contracture during the neodermis remodeling process, due to their superior stiffness. To hasten neodermis growth after implantation, tissue engineering strategies aim at cellularizing the dermis compartment prior to implantation with either fibroblasts or endothelial cells. Pre-vascularization has been shown to improve the take of the STSG and the reperfusion of the dermal bed. This aids the oxygenation of the zone and the removal of waste. The presence of fibroblasts serves to shorten the migration time of fibroblasts from the recipient and to also promote the synthesis of the neo-ECM in the wound bed [26]. Nevertheless, once assembled, the final ECM is still far from its physiologic condition. By analyzing the composition of the bioengineered skin models, it is possible to highlight that they are characterized by a common denominator: cells are always embedded in exogenous matrices (i.e., not synthesized by fibroblasts). This can represent an issue, since native ECMs possess a specific arrangement of moieties, which regulates the cross-talk between fibroblasts and keratinocytes that ultimately leads to the formation of skin adnexa and skin appendages [16,17,18]. The in vitro fabrication of human bioengineered dermis composed of a fibroblast-assembled ECM incorporating a vascularized network may provide a means of overcoming the scarring process. In this regard, it has been shown that a tissue engineering strategy allows the control of the assembly of the ECM produced by fibroblasts. By modulating the process variables, it is possible to produce an engineered dermis possessing composition, organization, and signal presentation capabilities relatively similar to the native dermis [19,34,35,72,74,81]. This may led to different benefits in the scenario of skin regeneration: (i) To date, the neodermis takes at least two years to form; by introducing the reconstructed patients’ dermis in the wound bed, possessing the final architecture and composition at the moment of the implant, no further time will be required for neodermis formation; (ii) by controlling in vitro the degree of assembly of the ECM, it will possible to decrease the differences between repaired and healthy tissues; and (iii) the spatial organization and the functionality of ECM components is not compromised by external factors (e.g., chemicals or physical treatments) and communications among all cell types (e.g., fibroblasts, keratinocytes, nerve endings and macrophages) are correctly orchestrated.

## Figures and Tables

**Table 1:** Acellular dermal substitutes.

Product	Composition	Indications	FDA Status
ALLODERM^®^	Acellular human dermis– non crosslinked	Repair or replacement of damaged or inadequate integument tissue	HCT/P
DERMACELL^®^	Acellular human dermis– non crosslinked	Chronic non-healing wounds	HCT/P
DERMAMATRIX^®^	Acellular human dermis– non crosslinked	Soft tissue replacement Breast Reconstruction	Available through the Musculoskeletal Transplant Foundation which meets and exceeds the standards and regulations of the American Association of Tissue Banks (AATB) and the Food and Drug Administration (FDA)
SUREDERM^®^	Acellular human dermis– non crosslinked	Soft tissue replacement	HCT/P
OASIS^®^	Porcine acellular lyophilized small intestine submucosa– non crosslinked	Acute, chronic and burns wounds. It delivers growth factors to stimulate and cell migration angiogenesis	510(k)
PERMACOLL^®^	Porcine acellular diisocyanite -crosslinked	Full-thickness defects such as burns and for soft tissue reconstruction such as hernia repair	510(k)
EZ-DERM^®^	Porcine aldehyde cross-linked reconstituted dermal collagen	Partial-thickness burns	510(k)
INTEGRA^®^	Acellular Bovine type I collagen and chondroitin- 6-sulfate copolymer coated with a thin silicone elastomer - crosslinked	Deep partial thickness and full thickness burns	PMA (1996) 510(k) (2002)
BIOBRANE^®^	Ultrathin silicone as epidermal analog film and 3D nylon filament as dermal analog with type I collagen peptides	Partial-thickness burns in children; toxic epidermal necrolysis, paraneoplastic pemphigus and chronic wounds	510(k)
MATRIDERM^®^	Bovine non-crosslinked lyophilized dermis, coated with α-elastin hydrolysate	Full-thickness burns	510(k)
HYALOMATRIX^®^	Acellular non-woven pad of benzyl ester of hyaluronic acid and a silicone membrane– non crosslinked	Burns, chronic wounds.	510(k)

Adapted from [9,29,39,40]. FDA status (retrieved from FDA website): A preamendment device is one that was in commercial distribution before May 28, 1976, the date the Medical Device Amendments were signed into law. After the Medical Device Amendments became law, the classification of devices was determined by FDA classification panels. Eventually all Class III devices will require a PMA. However, preamendment Class III devices require a PMA only after FDA publishes a regulation calling for PMA submissions. The preamendment devices must have a PMA filed for the device by the effective date published in the regulation in order to continue marketing the device. The CFR will state the date that a PMA is required. Prior to the PMA effective date, the devices must have a cleared Premarket Notification 510(k) prior to marketing. Class III Preamendment devices that require a 510(k) are identified in the CFR as Class III and include the statement “Date premarket approval application (PMA) or notice of completion of product development protocol (PDP) is required. No effective date has been established of the requirement for premarket approval.”.

**Table 2:** Cellularized skin substitutes.

Product	Composition	Indications	Status
DERMAGRAFT^®^ (d)	Human cultured neonatal fibroblasts seeded on polyglactin scaffold	Treatment of diabetic foot ulcers, epidermolysisbullosa	PMA (2001)
TRANSCYTE^®^ (d)	Nylon mesh coated with bovine collagen and seeded with allogenic neonatal human foreskin fibroblasts	Full and partial thickness burns	PMA (1998)
ICX-SKN^®^ (d)	A fibrin matrix seeded with neonatal human fibroblasts	Deep dermal wounds	-
DENOVODERM^®^ (d)	Autologous fibroblasts in collagen hydrogel	Deep defect of the skin	In development, under clinical trials
HYALOGRAFT3D^®^ (d)	Based on estherified hyaluronic acid derivate with cultured fibroblasts and covered by a silicone membrane	Use in diabetic ulcer therapy has been reported	510(k)
APLIGRAFT^®^ (ft)	Bovine collagen matrix seeded with neonatal foreskin fibroblasts and keratinocytes	Treatment of various forms of epidermolysisbullosa Diabetic and venous ulcers	PMA Commercially available in the USA
TISSUETECH^®^ (ft)	Hyaluronic acid with cultured autologous keratinocytes and fibroblasts (Hyalograft 3D^®^ + Laserskin^®^)	Ulcers	-
PERMADERM^®^ (ft)	Autologous fibroblasts and keratinocytes in culture with bovine collagen and GAG substrates	Sever Burns	-
ORCEL^®^ (ft)	Type I bovine collagen matrix seeded with allogenic neonatal foreskin fibroblasts and keratinocyte	Donor sites in Epidermolysis Bullosa Fresh, clean split thickness donor site wounds in burn patients	PMA (2001)
DENOVOSKIN^®^ (ft)	Autologous fibroblasts in collagen hydrogel and autologous keratinocytes	Deep defect of the skin	In development, under clinical trials

D = composed of one layer (engineered dermis); FT = full-thickness, composed of engineered dermis and engineered epidermis. Adapted from [9,29,39,40]. FDA status: see caption in Table 1.
